# Editorial Comment: Risk Factors for Penile Fracture After Intralesional Collagenase Clostridium histolyticum in Peyronie's Disease

**DOI:** 10.1590/S1677-5538.IBJU.2024.9906

**Published:** 2024-03-18

**Authors:** Luciano A. Favorito

**Affiliations:** 1 Universidade do Estado do Rio de Janeiro Unidade de Pesquisa Urogenital Rio de Janeiro RJ Brasil Unidade de Pesquisa Urogenital - Universidade do Estado do Rio de Janeiro - Uerj, Rio de Janeiro, RJ, Brasil;; 2 Hospital Federal da Lagoa Rio de Janeiro RJ Brasil Serviço de Urologia, Hospital Federal da Lagoa, Rio de Janeiro, RJ, Brasil

Isaac Zucker 1, Sirpi Nackeeran 2, Sahaam Mirza 3, Thomas A Masterson 4

**Urology. 2024 Jan:183:117-120.**

**DOI: 10.1016/j.urology.2023.10.019 | ACCESS: 37949243**

## COMMENT

The treatment of Peyronie's disease (PD) is challenging. The paper of Zucker et al. (1) shows an important complication after the use of Intralesional Collagenase Clostridium histolyticum (CCh) in PD: Penile fracture. Sexual intercourse represented the main cause of PF in USA, Europe and Brazil because intercourse is generally associated with high-energy traumas (2). Recent papers showed that, the positions with the “man on top” and “doggy style” were considered the most severe, presenting greater association with urethral and bilateral lesions of the corpora cavernosa (2, 3).

In the present paper the authors studied retrospectively 1541 patients that received at least one injection of CCh for PD. Of them, 0.7% (11/1541) suffered corporal rupture occurred at a median of 8 days after CCh injection. The majority of fractures were secondary to spontaneous erections or sexual intercourse. Finally, six patients had their fracture repaired surgically while the remaining were managed conservatively. The authors concluded that intralesional CCh can lead to corporal rupture and the most fractures occurred within 2 weeks of CCh injections and were associated with sexual intercourse and spontaneous morning erections.
